# Prevalence of Primary Biliary Cholangitis in a Cohort of Primary Sjögren’s Syndrome Patients

**DOI:** 10.7759/cureus.24590

**Published:** 2022-04-29

**Authors:** Gonçalo A Santos, Mariana Brandão, Fátima Farinha

**Affiliations:** 1 Department of Internal Medicine, Hospital de Braga, Braga, PRT; 2 Clinical Immunology Unit, Centro Hospitalar Universitário do Porto, Porto, PRT

**Keywords:** autoimmune epithelitis, chronic liver disease, antimitochondrial autoantibodies, primary biliary cholangitis, sjögren's syndrome

## Abstract

Objective

To analyze the prevalence and clinical progression of primary biliary cholangitis (PBC) in patients with primary Sjögren's syndrome (pSS) and possible associations between biochemical and immunological features and the development of PBC.

Methods

We retrospectively reviewed a cohort of 115 pSS patients followed up in an outpatient clinic from 1987 to 2020, without a history of liver disease, and looked for the presence of PBC through analysis of several biochemical, immunological, and histologic characteristics.

Results

Twenty patients (17.4%) had chronic cholestatic liver biochemistry. After exclusion of extrahepatic liver disease by abdominal ultrasound, 13 of them were tested for antimitochondrial autoantibodies (AMA) detected by indirect immunofluorescence (IF), of which five tested positive, fulfilling the diagnostic criteria for PBC. Three of the five PBC patients and three of the eight chronic cholestasis AMA-negative patients were further investigated with liver biopsy, which showed features of PBC in all three PBC patients and in one of the chronic cholestasis AMA-negative patients, allowing for the diagnosis of AMA-negative PBC in the latter. The remaining two AMA-negative patients had liver histology compatible with autoimmune hepatitis and unspecific findings, respectively. Overall, six (5.2%) patients with pSS had AMA-positive PBC (n=5) or AMA-negative PBC (n=1). Comparing immunological characteristics between PBC and non-PBC patients, we found that PBC patients had a higher mean maximum erythrocyte sedimentation rate (ESR) during follow-up than patients without PBC. All PBC patients were treated with ursodeoxycholic acid (UDCA) and after treatment with UDCA, only one patient showed biochemical and clinical progression of PBC, with increasing alkaline phosphatase and total bilirubin levels, eventually progressing to cirrhosis.

Conclusions

Among patients with pSS, PBC had an overall prevalence of six of 115 (5.2%). Higher ESR was a feature associated with PBC patients. In our cohort, after initiation of UDCA treatment, PBC showed predominantly slow progress, with only one patient progressing to cirrhosis during follow-up.

## Introduction

Sjögren’s syndrome (SS) is a chronic systemic autoimmune disorder characterized by lymphoplasmacytic infiltrates of the exocrine glands, which results primarily in xerostomia and xerophthalmia but may also cause extraglandular manifestations [1-2]. Its prevalence varies across different regions and in a recent meta-analysis, the overall prevalence was estimated at 0.043% [3]. Women are more frequently affected than men, with an overall female-to-male ratio of approximately 10:1 [3-4].

SS is classified as primary (pSS) when it occurs alone or in association with organ-specific autoimmune diseases, such as autoimmune thyroiditis, or secondary (sSS), when the disease occurs in association with another systemic autoimmune disease such as systemic lupus erythematosus [2,5].

Primary biliary cholangitis (PBC), previously known as primary biliary cirrhosis, is the most common autoimmune liver disease reflected histopathologically by a chronic immune-driven injury to the small bile ducts [6-8].

Both diseases commonly occur together in what has been called chronic autoimmune epithelitis [9], and previous studies showed a prevalence of PBC in pSS as high as 9% [10-12]. Conversely, a previous study reported that 18 of 38 PBC patients had symptoms of SS [13].

In the present work, we aim to study the prevalence of PBC in a cohort of pSS patients, analyzing the biochemical, immunological, and histopathological features.

## Materials and methods

Patients

**Figure 1:**
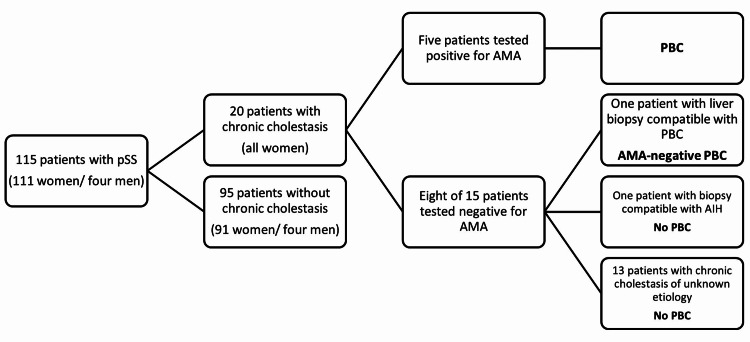
Prevalence of primary biliary cholangitis in patients with primary Sjögren’s syndrome AIH: autoimmune hepatitis; AMA: antimitochondrial autoantibodies; PBC: primary biliary cholangitis; pSS: primary Sjögren's syndrome

Patients were selected from the outpatient clinic database of the Clinical Immunology Unit, Centro Hospitalar Universitário do Porto, Portugal, which contains data from pSS patients followed up since 1987 and submitted to clinical, biochemical, and immunological reevaluation at least every six months. Only three patients were lost to follow-up (2.6%) and were included in the study until the year of loss to follow-up. All patients fulfilled the criteria for the diagnosis of pSS by the 2002 AECG classification criteria [14]. Patients with a history of alcohol abuse, hepatotoxic drug use, a known diagnosis of hepatitis B or C virus or HIV infection, or signs of metabolic or lymphoproliferative diseases were excluded. In total, 115 patients (111 women, 4 men, mean age 59.2 years ± 14.7) with a diagnosis of pSS were included in the study (Figure 1).

Study protocol

We reviewed all 115 database files in June 2020 and searched for the presence of chronic cholestasis, defined by the elevation of alkaline phosphatase (ALP) for more than six months [15], confirming its hepatic origin by the simultaneous elevation of gamma-glutamyl transpeptidase (GGT). We also registered if there was an elevation of alanine aminotransferase (ALT) and/or aspartate aminotransferase (AST), and bilirubin. Of those deemed to have chronic cholestasis, we investigated the result of the anti-mitochondrial autoantibody (AMA) test, measured by indirect immunofluorescence (IF), with a titer greater than 1/40 being considered a positive result, and searched for the presence of the more specific AMA-M2 subtype, measured by enzyme-linked immunosorbent assay (ELISA). We also searched for the presence of PBC-specific antinuclear antibodies (ANA) gp210 and sp100.

We registered hematological parameters of pSS patients, including erythrocyte sedimentation rate (ESR), immunoglobulin G (IgG) concentration, and the presence of anemia (hemoglobin concentration <12 g/dL), neutropenia (neutrophil count <1500 per microliter), and thrombocytopenia (platelet count <100.000 per microliter), as well as the presence of immunological parameters, including rheumatoid factor (RF), ANAs, Sjögren's syndrome autoantibodies A and B (SSA and SSB), hypocomplementemia, defined as a low C3 and/or C4 [16] and cryoglobulinemia. We also investigated the presence of extraglandular (systemic) involvement by searching for the presence of certain clinical features, i.e., arthritis, cutaneous vasculitis, Raynaud’s phenomenon, autoimmune thyroiditis (altered thyroid function with positive antithyroid autoantibodies), renal involvement (diagnosis of renal tubular acidosis, interstitial nephritis, or glomerulonephritis), and pulmonary involvement (presence of chronic cough and either abnormal findings on high-resolution computed tomography of the thorax or impaired pulmonary function tests).

Diagnosis of PBC

The diagnosis of PBC was made whenever chronic cholestasis was noted in the biochemical reevaluations together with a positive AMA test at a titer >1:40, after exclusion of extrahepatic causes of cholestasis or liver neoplasms by abdominal ultrasound, in agreement with the 2017 EASLD Clinical Practice Guidelines [17].

We also retrieved data from liver biopsies, performed after informed consent in six patients, and looked for histological signs of PBC or alternative diagnoses.

In both instances, we assumed the date of diagnosis of PBC as the date of onset of cholestasis.

Statistical analysis

We performed the univariate and bivariate descriptive analysis of pSS patients with and without chronic cholestasis and of pSS patients with and without PBC diagnosis, through the frequency statistics (absolute and relative) of categorical variables and through descriptive statistics of continuous variables: measures of location (mean and median), dispersion (standard deviation), and amplitude (minimum and maximum).

We used the student's t-test to compare the mean values of continuous variables and the chi-square test to evaluate the dichotomous variables. In cases where the assumptions of the chi-square were violated, we applied Fisher's exact test.

Whenever feasible, we analyzed the point and interval estimate of relative risk, considering a confidence interval of 95%. The significance level was set at α=0.05. Statistical analysis was performed using SPSS vs 22 (IBM Corp., Armonk, NY).

## Results

Of the 115 patients with pSS, 20 (17.4%) had chronic cholestatic liver disease defined by the elevation of ALP and GGT for more than six months (Figure 1). The main demographic, hematological, and immunological manifestations of patients with pSS and chronic liver cholestasis were compared with the 95 pSS patients without liver cholestasis (Table 1).

**Table 1:** Demographic, hematological, and immunological manifestations of pSS patients with chronic cholestasis compared with pSS patients without chronic cholestasis AMA: antimitochondrial autoantibodies; ANA: antinuclear antibodies; anti-La/SSB: autoantibodies directed against anti-La/SSB autoantigens; anti-Ro/SSA: autoantibodies directed against Ro/SSA autoantigens; ESR: erythrocyte sedimentation rate; PBC: primary biliary cholangitis; pSS: primary Sjögren's syndrome

	Chronic cholestasis (n=20)	No chronic cholestasis (n=95)	p
Demographic features			
Mean age, yrs	63.1 ± 15.1	58.3 ± 14.5	0.184
Female sex, no. (%)	20 (100)	91 (95.7)	1.000
Hematological data			
Maximum ESR, mm/h (mean ± SD)	67.0 ± 25.3	55.6 ± 28.4	0.100
Maximum IgG, mg/dL (mean ± SD)	2004.9 ± 778.1	1969.3 ± 1059.2	0.887
Anemia (Hb< 12 g/dL)	12 (60)	35 (36.8)	0.079
Neutropenia (<1500/µL)	3 (15)	18 (18.9)	1.000
Thrombocytopenia (<100.000/µL)	3 (15)	4 (4.2)	0.100
Immunological markers			
ANA	18 (90)	83 (87.4)	0.107
Anti-Ro/ SSA	17 (85)	80 (84.2)	1.000
Anti-La/ SSB	11 (55)	47 (49.5)	0.806
AMA	5/13 (38.5)	0	0.429
Rheumatoid factor	13/19 (68.4)	51/84 (60.7)	0.608
Hypocomplementemia	6 (30)	18 (18.9)	0.362
Cryoglobulinemia	4/16 (25)	15/75 (20)	0.736

Patients with chronic liver cholestasis were older (63.1 vs 58.3 years) and had a higher mean maximum ESR (67.0 vs 55.6 mm/h) during follow-up, in comparison with patients without chronic liver cholestasis, although none of those differences reached the significance level (Table 1). Anemia (60% vs 36.8%) and thrombocytopenia (15% vs 4.2%) were also more frequent in pSS patients with chronic cholestasis, again not reaching statistical significance.

Of the 20 patients with chronic cholestasis, after exclusion of extrahepatic causes of liver cholestasis by abdominal ultrasound, 13 were tested for AMA by IF. Of those, five had an AMA titer greater than 1/40, which established the diagnosis of PBC. Despite having a positive AMA test, three of those patients also underwent a liver biopsy, which allowed for the histologic staging of PBC. Two of the biopsies showed findings compatible with stage 1 PBC, with enlarged portal spaces with nests of inflammatory infiltrates comprised mainly of lymphocytes while the other one showed already bridging fibrosis compatible with stage 3 PBC.

Of the eight patients with chronic cholestasis but with a negative AMA test, three also underwent liver biopsy. One of the biopsies showed lymphocytic infiltrates confined to the portal tracts, compatible with stage 1 PBC, and accordingly a diagnosis of AMA-negative PBC was made. One patient had features of autoimmune hepatitis while one had unspecific findings. This last patient together with the five AMA-negative patients without a liver biopsy, and the seven chronic cholestasis patients not tested for AMA and without a liver biopsy were deemed to have cholestasis of unknown etiology (n=13).

Characterization of PBC patients

Overall, six (5.2%) patients with pSS were found to have AMA-positive PBC (n=5) or AMA-negative PBC (n=1). All of them were women. In half of them (n=3), the diagnosis of PBC was made before the diagnosis of pSS, in two, the diagnosis of PBC was made in the same year as the diagnosis of PBC and in one, the diagnosis of PBC was made after the diagnosis of pSS. Thus, in our cohort, the mean age at diagnosis of PBC was 45.5 years vs 46.6 years at diagnosis of pSS. In patients without PBC, the mean age at diagnosis of pSS was 50.9.

The mean follow-up of pSS patients with PBC diagnosis was 9.2 years (SD ± 7.9) compared to 8.4 years (SD ± 5.7) for pSS patients without PBC (Table 2).

**Table 2:** Characteristics of pSS patients with PBC compared to pSS patients without PBC ALP: alkaline phosphatase; AMA: antimitochondrial autoantibodies; AMA-M2: M2-subtype antimitochondrial autoantibodies; ANA: antinuclear antibodies; anti-La/SSB: autoantibodies directed against anti-La/SSB autoantigens; anti-Ro/SSA: autoantibodies directed against Ro/SSA autoantigens; ESR: erythrocyte sedimentation rate; GGT: gamma-glutamyl transpeptidase; gp210: anti-nuclear glycoprotein 210 autoantigen; PBC: primary biliary cholangitis; pSS: primary Sjögren's syndrome; sp100: anti-nuclear sp100 autoantigen

	Patients with PBC (n=6)	Patients without PBC (n=109)	p
Demographic features			
Mean age, yrs	55.8 ± 15.9	59.4 ± 14.7	0.569
Female sex, no. (%)	6 (100)	105 (96.3)	1.000
Mean follow-up time, years	9.2 ± 7.9	8.4 ± 5.7	0.833
Liver involvement			
Elevated ALP	6 (100)	14 (12.8)	<0.001
Elevated ALP>1.5x normal	3 (50.0)	5 (4.6)	<0.004
Elevated GGT	6 (100)	17 (15.6)	<0.001
Elevated aminotransferases	3 (50.0)	9 (8.3)	0.015
Elevated total bilirubin	4 (66.7)	2 (1.8)	<0.001
Hematological data			
Maximum ESR, mm/h (mean ± SD)	70.8 ± 13.2	56,9 ± 28.6	0.050
Maximum IgG, mg/dL (mean ± SD)	1840.2 ± 819.4	1938.1 ± 1024.8	0.738
Immunological markers			
AMA	5 (83.3)	0/8 (0)	<0.001
AMA-M2	5 (83.33)	1/10 (10)	0.008
ANA	5 (83.33)	96 (88.07)	1.000
sp100	1/5 (20)	1/5 (20)	1.000
gp210	1/5 (20)	1/5 (20)	1.000
Anti-Ro/SSA	4 (66.7)	93 (85,3)	0.237
Anti-La/SSB	4 (66.7)	58 (49,5)	0.679
Rheumatoid factor	2/5 (0.4)	98 (63.3)	0.364
Hypocomplementemia	2 (33.3)	22 (20.2)	0.603
Cryoglobulinemia	0/3 (0)	19/88 (21.6)	0.491

All six PBC patients had increased GGT levels together with increased ALP, and half of them also had an elevation of aminotransferases. Four PBC patients experienced a rise in total bilirubin levels above normal during follow-up, but only one sustained those levels over time. The mean maximum ESR value was higher in patients with PBC (70.8 mm/h) than in patients without PBC (56.9 mm/h) (difference of 13.9 mm/h; 95% confidence interval (CI), 0.02 to 27.94 mm/h). There was no statistically significant difference in the mean maximum IgG value and in the presence of ANA between patients with PBC and without PBC. Similarly, there was no significant difference in the presence of rheumatoid factor, hypocomplementemia, anti-Ro (SSA), anti-La (SSB), or cryoglobulinemia.

Certain extraglandular features were more frequent in pSS patients with PBC than in those without PBC, particularly autoimmune thyroiditis (33.3 vs 11.9%, p=0.175) and lung involvement 33.3 vs 5.5%, p=0.055), the latter on the verge of statistical significance (Table 3).

**Table 3:** Extraglandular features of pSS patients with and without PBC pSS: primary Sjögren's syndrome; PBC: primary biliary cholangitis

	Patients with PBC (n=6)	Patients without PBC (n=109)	p
Arthritis	4 (66.7)	63 (57.8)	1.000
Autoimmune thyroiditis	2 (33.3)	13 (11.9)	0.175
Cutaneous vasculitis	0 (0)	14 (12.8)	1.000
Kidney involvement	0 (0)	7 (6.4)	1.000
Lung involvement	2 (33.3)	6 (5.5)	0.055
Raynaud phenomenon	1 (16.7)	36/105 (34.3)	0.622

At diagnosis of PBC, all patients reported symptoms of fatigue, and only one patient reported symptoms of pruritus. All patients were treated with ursodeoxycholic acid (UDCA), with a total dose ranging from 500 mg to 1000 mg divided into two daily doses (Table 4). After initiation of UDCA, three patients maintained a stable cholestatic pattern while two patients showed complete recovery of normal liver biochemistry. One patient showed clinical and laboratorial progression to cirrhosis despite the initiation of UDCA, with worsening of biochemical cholestasis and total bilirubin and development of portal hypertension.

**Table 4:** Autoantibodies titers, pathological staging, and response to treatment with UDCA of patients with PBC †Patient died during follow-up; AMA: antimitochondrial autoantibody; AMA-M2: M2-subtype antimitochondrial autoantibodies; gp210: anti-nuclear glycoprotein 210 autoantigen; sp100: anti-nuclear sp100 autoantigen; UDCA: ursodeoxycholic acid; PBC: primary biliary cholangitis

Patient	Age (years)	AMA titer	AMA-M2 titer	PBC-specific antibodies	Liver biopsy stage	UDCA dose (mg)	Response to UDCA treatment
1	62	-	-	-	1	500	Stable cholestasis
2	58	>1/128	179	-	Not performed	750	Stable cholestasis
3	71	1/1280	132.5	-	Not performed	750	Improved Cholestasis
4†	27	>1/128	165	gp210	1	750	Improved Cholestasis
5	67	>1/128	168	-	3	1000	Progression to cirrhosis
6	50	1/320	143	sp100	1	500	Stable cholestasis

Despite the improvement of cholestasis after initiation of UDCA, a 27-year-old pSS patient with PBC died of leptospirosis approximately seven years after the diagnosis of PBC. She also had neuromyelitis optica, with a positive aquaporin-4 antibody.

The patient with AMA-negative PBC didn’t develop positive AMA during a follow-up of one year after the diagnosis of PBC. Similarly, all 13 patients with cholestasis of unknown etiology remained AMA-negative during a mean follow-up of 8.8 years (range 1-28).

Analysis of PBC-specific antibodies

All patients with AMA in their sera had elevated ALP. The AMA titers ranged from 1/128 to 1/1280 (Table 4). Six of 16 patients tested positive for the M2 subtype of mitochondrial autoantibody (Table 2). Of those, only one patient didn't have chronic cholestasis and PBC diagnosis, and during follow-up, this patient did not develop biochemical cholestasis or symptoms of PBC. PBC antinuclear antibodies sp100 and gp210 were identified in 3/10 patients (Table 2). One patient with sp100 and one patient with gp210, both with PBC diagnosis, had, respectively, an AMA titer of 1/320 and >1/128 (Table 4). A third patient was positive for both sp100 and gp210, had chronic cholestasis, negative AMA but, interestingly, the liver biopsy showed only unspecific findings that did not confirm the diagnosis of PBC. The AMA-negative patient was negative for AMA-M2, sp100, and gp210.

## Discussion

In our study, the diagnosis of PBC was established in six patients, including five patients with chronic elevation of serum ALP and a positive AMA test and one patient with chronic elevation of serum ALP and a liver biopsy with compatible histology, despite a negative AMA test. That translates into an overall PBC prevalence of 5.2% in our cohort of pSS patients, which is similar to previous studies. Whaley et al. in the 1970s were the first to include AMA as a marker of autoimmune liver disease and found a frequency of positive AMA associated with a cholestatic pattern in 6.0% of 50 pSS patients [18]. Then, in the 1990s, Skopouli et al. [10] detected AMA in 6.6% of a large sample of 300 pSS patients, of which 80% had histological features compatible with stage I PBC, while Lindgren et al. [11] found that 9.1% of 55 pSS patients had positive AMA together with liver cholestasis. More recently, Ramos-Casals and colleagues [19] found a prevalence of PBC of 3,4% in 475 patients with pSS, second only to chronic HCV disease, and Hatzis et al. [12] found a prevalence of PBC of 6.6% in a large cohort of 410 patients. Karp and colleagues [20] estimated a lower prevalence of PBC (1%) in a series of 202 patients in 2010.

Our study, therefore, adds to the pool of evidence that PBC is a prevalent autoimmune hepatic disease in pSS patients by following a cohort of 115 pSS patients for as long as 33 years.

Another significant finding was the higher mean ESR value in pSS patients who developed PBC in comparison to patients that didn’t develop PBC, which is identical to the study by Ramos-Casals [19]. We didn’t find the same trend in other parameters like maximum IgG, presence of RF, or hypocomplementemia in patients with PBC vs without PBC, perhaps because of the retrospective nature of our study or because the sample size was underpowered for that analysis. Moreover, different from the study by Ramos-Casals et al. [19], in our study, PBC was the most frequent liver disease and we found no cases of chronic viral hepatitis.

Excluding the six PBC patients and the patient with features of AIH in the liver biopsy, 13 patients (11.3%) had sustained elevation of ALP and, accordingly, chronic cholestasis of unknown etiology. This is also in accordance with previously reported data [10-12,20]. This proportion may have several explanations. On the one hand, we may have missed some diagnoses because of the testing of AMA by indirect immunofluorescence. Several studies have pointed out that if more sensitive methods like ELISA were used, the detection of antibodies increased, and consequently, the prevalence of PBC also increased [10,21-22]. On the other hand, some of the patients might still be in an earlier phase of liver disease. One patient without cholestasis was AMA-M2 positive and while it may have been a false positive, a previous study has shown that AMA-M2 positive patients with signs of liver involvement have a higher risk of developing symptomatic PBC [23]. This mandates a regular follow-up of liver biochemistry in patients with pSS to achieve an early diagnosis of PBC and start effective treatment with UDCA. Lastly, the retrospective design of our study didn’t allow us to test for antibodies for concurrent diagnoses like AIH, which may have also increased the proportion of idiopathic cholestasis.

We found one patient with AMA-negative PBC out of six cases of PBC. Previously published studies state that approximately 5% of patients with PBC do not have detectable AMA [24]. Again, the lack of a more sensible confirmatory test than IF may have impacted the identification of AMA and, therefore, of potential PBC cases. Previous studies describe alternative techniques, including ELISA and immunoblot analysis, which increase sensitivity for the detection of the major immunoreactive mitochondrial autoantigens (M2 subtype) [25], and therefore may diminish the proportion of AMA-negative PBC cases. Additionally, several types of ANAs have been associated with PBC, including the sp100 and gp210 antigens and recently two novel PBC autoantigens, kelch-like 12 (KLHL12) and hexokinase 1 (HK1), have been discovered [26]. In our study, the AMA-negative PBC patient also tested negative for the M2 subtype and for the sp100 and gp210 antigens by ELISA but in other chronic cholestasis patients in our cohort, those tests weren’t ordered by the assistant physician, which may have impacted the diagnosis of PBC. Again, this is a contingency of the retrospective design of our study.

Several characteristics distinguish our study from prior investigations. First, to our knowledge, this is the first study of PBC prevalence in a Portuguese cohort of pSS patients, in contrast with previous studies that analyzed PBC prevalence in pSS patients of Greece, Sweden, Spain, and the USA [10-11,19-20], with a somewhat different genetic background. All patients fulfilled the AECG criteria for the diagnosis of pSS and the diagnosis of the PBC cases was made according to the newest criteria by the EASLD.

Limitations of the study, besides those previously stated, include those common to retrospective studies conducted over a prolonged period of time. It is possible that there have been changes in the diagnostic approach, workup studies, and treatment strategies in patients with pSS and PBC over time.

More studies are needed to better clarify the differences, if any, between AMA-negative patients from AMA-positive patients, or if AMA-negative cases are only due to a lack of sensible diagnostic techniques.

## Conclusions

In conclusion, our study estimated a 5.2% prevalence of PBC in a cohort of 115 pSS patients. The higher ESR was a distinctive feature of PBC patients, and in all but one patient, the disease showed slow progress after the initiation of treatment with UDCA. There was a considerable proportion of patients with chronic cholestasis of unknown etiology, without distinctive features in comparison with the rest of the cohort, possibly representing a liver disease yet to be diagnosed or an incidental finding that nonetheless indicates a regular follow-up.
